# Expression-based Pathway Signature Analysis (EPSA): Mining publicly available microarray data for insight into human disease

**DOI:** 10.1186/1755-8794-1-51

**Published:** 2008-10-20

**Authors:** Jessica D Tenenbaum, Michael G Walker, Paul J Utz, Atul J Butte

**Affiliations:** 1 Stanford Medical Informatics, 251 Campus Drive MSOB x215, Stanford, CA 94305, USA; 2 Stanford University School of Medicine, Department of Medicine, Division of Immunology and Rheumatology, CCSR Building, Room 2215, Stanford, CA 94305, USA; 3 Stanford University School of Medicine, Department of Pediatrics, Stanford, CA 94305, USA; 4 Duke Translational Medicine Institute, PO Box 17969, Durham, NC 27715, USA

## Abstract

**Background:**

Publicly available data repositories facilitate the sharing of an ever-increasing amount of microarray data. However, these datasets remain highly underutilized. Reutilizing the data could offer insights into questions and diseases entirely distinct from those considered in the original experimental design.

**Methods:**

We first analyzed microarray datasets derived from known perturbations of specific pathways using the samr package in R to identify specific patterns of change in gene expression. We refer to these pattern of gene expression alteration as a "pathway signatures." We then used Spearman's rank correlation coefficient, a non-parametric measure of correlation, to determine similarities between pathway signatures and disease profiles, and permutation analysis to evaluate false discovery rate. This enabled detection of statistically significant similarity between these pathway signatures and corresponding changes observed in human disease. Finally, we evaluated pathway activation, as indicated by correlation with the pathway signature, as a risk factor for poor prognosis using multiple unrelated, publicly available datasets.

**Results:**

We have developed a novel method, Expression-based Pathway Signature Analysis (EPSA). We demonstrate that ESPA is a rigorous computational approach for statistically evaluating the degree of similarity between highly disparate sources of microarray expression data. We also show how EPSA can be used in a number of cases to stratify patients with differential disease prognosis. EPSA can be applied to many different types of datasets in spite of different platforms, different experimental designs, and different species. Applying this method can yield new insights into human disease progression.

**Conclusion:**

EPSA enables the use of publicly available data for an entirely new, translational purpose to enable the identification of potential pathways of dysregulation in human disease, as well as potential leads for therapeutic molecular targets.

## Background

Publicly available data repositories facilitate the sharing of an ever-increasing amount of microarray data. However, the datasets in these repositories typically remain highly underutilized following the analysis for which they were originally intended. This situation is unfortunate because reusing the data can offer insights into pathways and diseases entirely distinct from those considered in original experimental designs.

In order to take full advantage of the data in the public domain, new ways of using and integrating the information will be required. To meet this need, we have developed a statistical method for pattern matching between highly disparate sources of data. Our approach compares gene expression alterations in human disease with expression patterns in publicly available collections, or compendia, of microarray data. These datasets contain information on changes in gene expression in response to perturbations of cells, such as stimulation with a small molecule or alterations due to a transgenic mutation. If similarities can be detected between the changes in gene expression in diseased versus normal tissues, and those changes observed by perturbing known pathways, these observations may provide new information regarding pathways that are potentially affected in disease. They also provide new insights into human disease and possible therapeutic interventions. Specifically, our method is useful for hypothesis generation in two important areas: (i) identifying pathways undergoing dysregulation in disease; and (ii) relating pathway activation to clinically relevant observations.

Although there are many knowledge-based resources (e.g. GO, the Gene Ontology) that can be used to determine the significance of genomic findings, relatively few investigators have pursued a data-driven approach for generating clinical or mechanistic hypotheses. Bild et al. used supervised machine learning classifiers to calculate a probability score for the presence or absence of specific oncogenic mutations [1]. The study showed that clustering patients by probability score across multiple mutations identified groups with significantly divergent prognoses. Crucial to the success of this approach was a strong expression signal and a sufficient number of replicates from which the classifier could learn. Unfortunately, machine learning algorithms are known to over-fit when given sparse training data, which is often the case with microarray experiments [2].

Recognizing that a novel approach was needed for pattern matching among noisy and disparate datasets, Lamb et al. created the Connectivity Map, which compares a query signature to a compendium of expression profiles using a non-parametric approach [3]. The main weakness to this approach is that it lacks a statistical method for determining the significance of observed connections.

An important question is whether a data-driven approach can use knowledge gained in the analysis of one dataset to aid the analysis of other microarray data. To address this question, we developed a method for finding similarity between disparate and noisy gene expression datasets. The method also calculates the statistical significance of its results. We applied it by comparing gene expression profiles from samples of human disease with profiles generated by known perturbations to cells. Our work shows that the method is robust to noise, sparse data, and missing values, and can use data from different microarray platforms and non-human species to draw conclusions about human disease. Our method is better at differentiating prognosis based on individual pathway activation than the approach employed by Bild et al. for a human ovarian cancer dataset. In addition, we show that our method can be applied to a small-molecule perturbation dataset to suggest potential therapeutic leads. Finally, we demonstrate that the method may be applied even across datasets from divergent species to yield findings that are suggestive of target pathways for therapeutic intervention.

## Results

**Figure 1:**
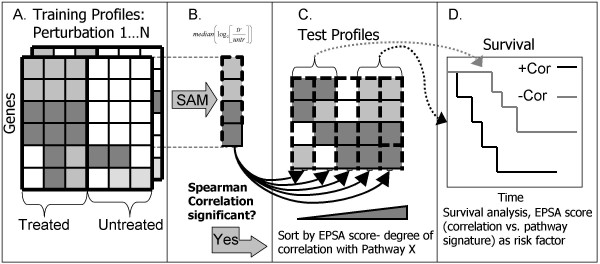
**Methodology for application of EPSA. **A. For each perturbation in the training compendium, replicate log_2 _expression of treated vs. untreated cells were analyzed. B. For each perturbation, Significance Analysis of Microarrays (SAM) [4] software was applied to identify genes with significantly altered expression. Median values among the replicates of log_2_(treated/untreated) were used to represent genes in a signature. C. These values were correlated with test profiles. A false discovery rate was calculated for each perturbation, for the average level of correlation with disease profiles. D. For those signatures with a statistically significant level of correlation, survival analysis was performed using the EPSA score, or the degree of signature correlation, as a factor influencing survival. Survival analysis was carried out using the *survival *package in R.

We used a robust, non-parametric measure of correlation to develop a general statistical method for reusing publicly available microarray data. The purpose of the method is to generate clinically relevant hypotheses regarding human disease. It employs the widely used SAM software package to define "pathway signatures" from known molecular perturbations [4]. The term "pathway signature" refers to the specific pattern of differential gene expression that was observed upon experimental perturbation of a given pathway. It is represented by the log_2 _of the fold change for the list of differentially expressed genes in the perturbed versus the non-activated state. Extracting this subset of genes is necessary in order to pinpoint the genes at play in the specific pathway of interest, and to eliminate other factors such as cell type could dominate any correlative signal detected. Our method can then assess the degree of statistical similarity between gene expression profiles in human disease and these pathway signatures, which in turn can be used as a measure of pathway activation. We are then able to evaluate pathway activation as a risk factor for patient survival. We call this approach Expression-based Pathway Signature Analysis (EPSA). Figure 1 provides an overview of EPSA.

### Application to existing, established datasets

#### EPSA correctly identifies pathway mutations in cancer data

**Figure 2:**
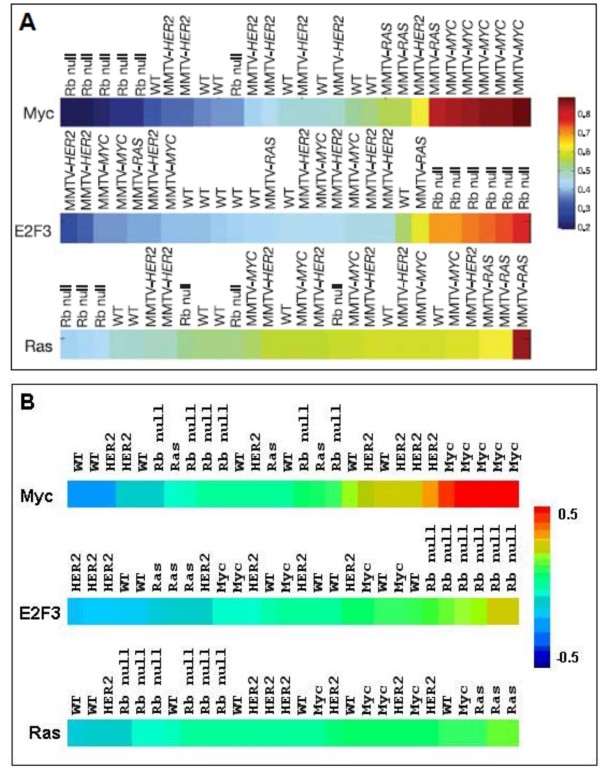
**Positive control validation for accuracy and specificity.** Gene expression profiles were used to compare accuracy and specificity of signatures from Bild's study (panel A, used with permission) and EPSA (panel B). The profiles were derived from mammary tumors in mice transgenic for the MMTV-MYC, MMTV-HRAS or MMTV-HER2 oncogenes, from murine tumors dependent on loss of Rb, or from seven samples of normal murine mammary tissue. The predicted probability of Myc, E2F3 and Ras activity in these tumors was sorted from low (blue) to high (red), and displayed as a color bar. Both methods correctly ranked the appropriate test cases as highest scoring (whether probability or correlation) for the respective mutations. Myc gave the strongest signal in both methods, followed by Rb null and Ras, in that order. (Loss of Rb causes an increase in E2F3 expression.)

Bild et al. trained Bayesian regression models on a training set derived from replicates of five different oncogenic mutations. These predictors were able to assess the relative probability of pathway deregulation in tumors and cell lines for these five affected pathways. The authors then demonstrated that pathway prediction for positive control samples tended to reflect the molecular basis for tumorigenesis. This tendency was indicated by probability scores derived from their method of regression analysis [1]. Positive controls were expression patterns from mammary tumors from mice carrying transgenic copies of the oncogenes used in the training set or, in the case of Rb null animals, knockouts of an inhibitor of E2F3 [5]. Figure 2A shows results for positive controls presented by Bild et al., and Figure 2B shows results for a similar analysis using EPSA correlation scores instead of a regression probability score. EPSA demonstrates a similarly high level of accuracy in identifying the actual underlying mutation in positive controls.

#### EPSA prognosis prediction outperforms that of Bild et al.'s more complex machine learning approach

**Figure 3:**
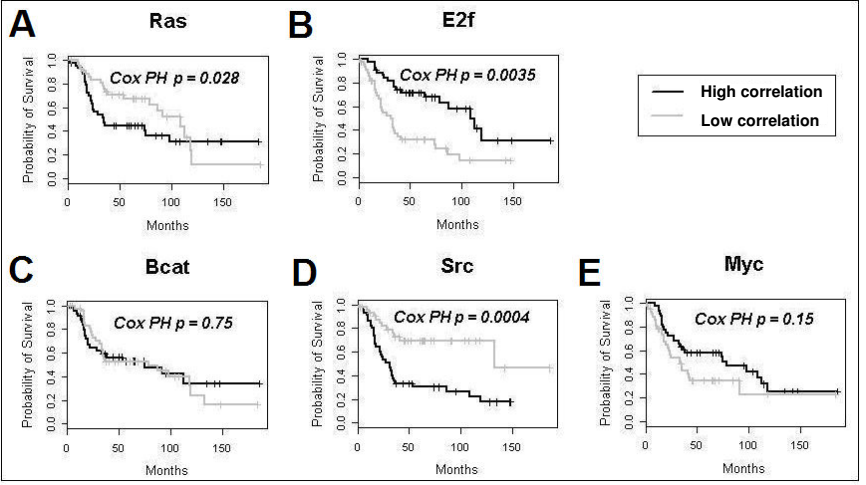
**Kaplan-Meier analysis for individual pathway activity as a factor for survival in ovarian cancer.** The patient cohort was from Bild et al. [1] Results from the Src pathway using Bild's method were not significant when corrected for multiple hypothesis testing (*p *= 0.04), while the *p*-value generated using EPSA and Cox proportional-hazards was highly significant (p = 0.0004). Note that although the EPSA curves show only the extreme tertiles of subjects, Cox proportional-hazards analysis was performed across all subjects in order to be directly comparable with the results in Bild et al.

Figure 3 shows Kaplan-Meier survival curves for an ovarian cancer patient cohort (*n *= 135). The curves are based on EPSA correlation scores for each of the five oncogenic mutations performed by Bild et al. (see Methods). They show the two extreme tertiles of patients: namely, the third of patients with the highest degree of correlation and the third with the lowest degree of correlation. *p*-values are provided for Cox proportional-hazards analysis across all patients (described in Methods). These results may be compared with those in Supplementary Figure 4 of the Bild et al. paper. Mutations in the Myc and E2F3 pathways are roughly comparable as factors influencing survival in both methods. Mutations of the Ras and β-catenin pathways had low *p*-values in the EPSA and Bild analyses, but neither were significant when corrected for multiple hypothesis testing. Importantly, results of the Src pathway using Bild's method were not significant when corrected for multiple hypothesis testing (*p *= 0.22), while the *p*-value generated using EPSA was highly significant at 0.002 after Bonferroni correction. One major finding of the paper by Bild et al. was that clustering patients into six groups based on all five mutations together gave a more significant differential prognosis. Here, too, EPSA differentiated survival to a statistically significant degree, (Kaplan-Meier *p*-value < 0.0093 [not shown]).

**Figure 4:**
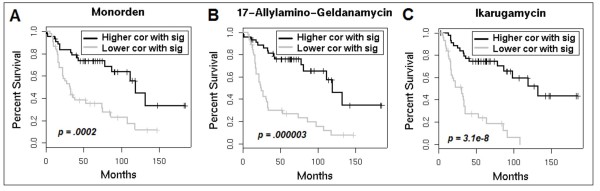
**Survival analysis for Connectivity Map perturbagen correlation as a risk factor.** Kaplan-Meier survival curves represent the top and bottom tertiles of patients with respect to correlation with the perturbagen signature. Black curves represent patients with higher correlation with the perturbagen signature, while the gray curve represents a lower level of correlation.

#### EPSA detects statistically lower correlation in disease profiles with Connectivity Map perturbagens

The Connectivity Map is a reference collection of expression profiles generated by stimulation of human cultured cells with a number of small molecules, along with an algorithmic method for data mining through pattern matching. One goal of the approach was to detect functional connections between disease and drug action. Of note, the authors showed that connections could be detected even across cell types. Of the thirty-two Connectivity Map perturbagens for which we were able to generate pathway signatures (see Methods), none showed a significantly higher degree of correlation with tumor expression profiles from the Bild et al. ovarian cancer cohort than randomly permuted signatures. However, twenty-one perturbagens showed significantly lower levels of correlation with disease data [see Additional file 1]. That is, disease tissue profiles tended to show lower correlation with the pathways activated by these perturbagens than would be expected by random chance.

#### EPSA predicts differential prognosis in cancer patients based on projected activation level of small-molecule pathways

For the twenty-one molecules with a lower degree of correlation with patient profiles from the ovarian cancer cohort, we performed survival analysis using the degree of correlation between the signature and the tumor expression profile as a factor influencing survival. After Bonferroni correction, five of the perturbagen signature correlation values were determined to be significant factors influencing survival. The directionality of the effect for two of them (LY294002 and Trichostatin Aa) was opposite to what would be intuitive or helpful therapeutically, but of particular interest were results for the remaining three compounds: monorden, 17-allylamino-geldanamycin (17AAG), and ikarugamycin (Cox proportional analysis *p*-values: 2 × 10^-4^, 3 × 10^-6^, and 3 × 10^-8 ^respectively; Figure 4). For these drugs, lower correlation with the drug signature corresponded to a lower rate of survival. Higher correlation with the drug signature corresponded to a higher rate of survival, though paradoxically, this higher correlation is also closer to the average expected correlation between the patient samples and the pathway signature. These results suggest that, for any one of these three small molecules, the corresponding pathway may be dysregulated in ovarian cancer, and that treatment with the drug could induce a beneficial drug signature. Of course, additional experiments would be required to tease out the actual underlying molecular mechanisms, and whether this proposed explanation would in fact play out with actual stimulation of ovarian tumor cells using these known therapeutic molecules.

### Application to a divergent species dataset

#### Pathway signature determination from compendium data

Based on clinical relevance and availability of outcome data, we chose B cells as a model cell on which to test our approach. Gene Expression Omnibus series GSE2050 provides time course data in which gene expression profiles from mouse B cells stimulated with 33 different ligands were compared to gene expression profiles of unstimulated B cells [6,7]. We used this data as the training compendium for EPSA. We used SAM [4] to identify pathway signatures of differentially expressed genes for each of the 33 ligands in the compendium dataset. We then sought to confirm that these pathway signatures would show a statistically significant degree of correlation with profiles generated through activation of the same pathways in other experiments, and on other microarray platforms.

#### EPSA identifies correlation patterns in positive controls from mouse B cells

GEO dataset GSE1014 (no associated PubMed ID) was generated by stimulating mouse B cells with a subset of ligands that had also been used in GSE2050. We used it as a positive control to confirm that correlations between test set profiles and corresponding ligand signatures derived from the compendium were detectable and differentiable. The resulting correlations, averaged among test replicates, are shown in Additional file 2A. In three out of the four test cases, the ligand used to stimulate the cells in the test set was correctly re-identified as the most highly correlated compendium profile. One test stimulation, lipopolysaccharide (LPS), was misidentified as CD40 ligand (CD40L), but LPS was a close second. In summary, our method demonstrated good sensitivity for detecting similarity between test profiles in mouse B cells and corresponding profiles derived from the mouse B cell compendium data.

#### EPSA detects correlation patterns in positive controls from human B cells

Applying our method to human B cells introduced additional complexity, in that the genes from the compendium mouse arrays had to be translated to their human orthologs and then translated again to the probes used on the human array platform. This was accomplished with NCBI's HomoloGene mapping (see Methods for details). Our method was designed to account for the fact that many genes might not be translated because of platform differences or a lack of orthologs. Note that these datasets were generated in an independent laboratory. All four stimulations in human B cells included Anti-Ig (AIG), and correlation with the AIG pathway signature for all four samples was high [see Additional file 2B]. In addition, CD40L correlation was high for the first two samples, which included CD40L, but lower for the second two samples, which did not include it. With the exception of IL4 stimulation in one sample, which did not demonstrate high correlation with the IL4 compendium profile, these results show that our method has good (though not perfect) sensitivity for detecting similarities between profiles, even when comparing across platform and species, and at different time points. Potential reasons for not detecting IL4 include one-time experimental or biological variability, since the test case represented only a single microarray. Interestingly, the next sequentially labeled experiment in the series in theory applied only AIG, but this array *did *show a higher degree of correlation with IL4. Without replicates, human error in the form of mislabeling cannot be ruled out.

#### Application of EPSA to diffuse large B cell lymphoma on cDNA microarrays

Having confirmed a reasonable level of sensitivity for correlation detection in positive controls, we next applied EPSA to microarray data from human samples of diffuse large B cell lymphoma (DLBCL), a B cell malignancy. The goal was to determine which pathways, if any, are activated in this disease, and if their activation has implications for survival. Using a dataset comprising gene expression profiles from 240 DLBCL patients [8], a number of the compendium ligands showed positive correlation with disease profiles [see Additional file 3].

To assess the significance of these correlations, we computed a false discovery rate (FDR) through permutation analysis (described in Methods). We identified four ligands with significant *q*-values: AIG, CD40, CPG, and LPS, with *q*-values of < 0.001, 0.08, 0.05, and 0.11 respectively [see Additional file 4].

**Figure 5:**
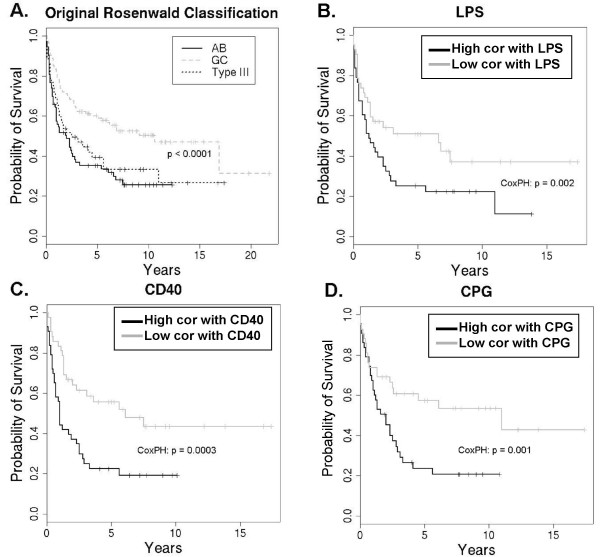
**Kaplan-Meier survival analysis comparing results from Rosenwald and EPSA.** A. Kaplan-Meier survival curve from Rosenwald, in which GC type diverges from the other subtypes (*p *< 0.0001), but AB and Type III do not diverge significantly (*n *= 240) [8]. B-D. Kaplan-Meier curves for activated B cell-like and type III DLBCL (*n *= 125) separated based on degree of correlation with LPS, CD40, and CPG signatures respectively. Patients in these plots are only those in the lower two curves from A; the GC subtype was excluded from analysis. Survival curves for the most- and least-correlated tertiles (*n *= 125/3 ≈ 42 each) diverge significantly, in contrast to that of the AB vs. type III distinction. Cox proportional-hazards *p*-value is shown for the subset of the 125 AB and type III patients.

We next performed survival analysis on the cohort of DLBCL patients using the level of correlation with the different signatures as factors influencing survival. The original study used unsupervised hierarchical clustering to group patients into three subtypes of DLBCL: germinal center-like (GC), activated B cell-like (AB), and type III. Of these subtypes, GC patients tended to have the best survival (*p *< 0.0001). AB and type III patients had poorer prognoses, but were statistically indistinguishable from each other using this methodology (Figure 5A).

We performed Cox proportional-hazards analysis on the same data to evaluate the EPSA score for the four statistically significant ligands listed above as potential factors influencing survival. Cox proportional-hazards analysis showed that AIG correlation was not a significant factor for survival, while the other three were significant, with Bonferroni corrected *p*-values for LPS, CD40, and CPG of 0.004, 0.0008, and 0.004 respectively (Figure 5B, 5C, and 5D). Toll-like receptor 4 (TLR4), Toll-like receptor 9 (TLR9), and CD40 are the receptors to which LPS, CPG, and CD40 ligand respectively bind to activate cells. Therefore, statistically significant, positive correlations with a signature found in TLR4, TLR9, and CD40 pathway activation were each associated with poor prognosis in diffuse large B cell lymphoma. As discussed below, a number of previous studies have demonstrated results that are in keeping with these findings.

Even more interesting are the results when this analysis is performed on activated B cell-like and type III, the two disease subtypes that were indistinguishable in the original study: Figure 5A shows that the AB and type III subtypes are statistically indistinguishable (*p *= 0.45). We analyzed these subtypes only and grouped by level of correlation (high, medium, or low) with pathway signatures for LPS, CD40, or CPG as described above. The results were the Kaplan-Meier curves shown in Figure 5B, 5C, and 5D. The curves represent the two extreme tertiles of patients, or the highest versus lowest degree of correlation. Cox proportional-hazards analysis across this entire subset of patients yielded Bonferroni corrected *p*-values of 0.008, 0.001, and 0.004 respectively for LPS, CD40, and CPG. The distinction being made is therefore independent from that of the GC, AB, type III system established in the original study [8].

## Discussion

We have presented a method that allows the use of publicly available microarray data to gain insight into human biology and disease. Our method can even be used on datasets generated in a model organism. The method can equal and even outperform more complex and labor-intensive methods when applied to established data. Furthermore, its results can be statistically validated through calculation of false discovery rate. In one example shown here, an existing dataset generated in order to gain insight into pathways of activation in mouse B cells provided relevant prior knowledge to facilitate novel insights and hypothesis generation in human cancer. Using publicly available microarray data from murine cells, we have shown that a pattern of differential prognosis emerges from assessment of similarity with known pathway signatures. Moreover, this pattern is reproducible despite the fact that test data were generated using entirely different microarray platforms and species from those used in the generation of training data. While this knowledge has direct implications for difficult decisions regarding aggressive therapeutic interventions in cancer treatment, the method is generalizable to numerous other clinical insights and discoveries. We assert that public biological data repositories are a rich and largely untapped resource for translational biomedical research.

Bild et al. demonstrated that pathway prediction in positive control samples, as indicated by probability scores, tended to reflect the molecular basis for tumorigenesis. Similarly, EPSA showed that pathway prediction for these samples, as indicated by Spearman rank correlation, also reflected underlying molecular mechanisms. Also consistent between the two methods is the relative strength of signal between the different mutations, with Myc most easily distinguished by both, and Ras the least distinguishable. Applying EPSA to the dataset provided by Bild, et al. yielded results that were comparable to the original results, which came from more a specialized and involved analysis.

With respect to application to the Connectivity Map data, increased patient profile correlation with ikarugamycin, 17-allylamino-geldanamycin, and monorden all resulted in better patient prognosis. Ikarugamycin is an antibiotic that inhibits oxidized low-density lipoprotein uptake [9]. It has also been implicated in the inhibition of clathrin-coated pit mediated endocytosis [10]. A number of related antibiotic compounds play a useful role in cancer therapy [11,12]. More specifically, the macrolide class of antibiotics, to which ikarugamycin belongs, can reverse drug resistance *in vitro *and *in vivo *[13].

17-allylamino-geldanamycin (17AAG) and monorden are both inhibitors of heat shock protein 90 (Hsp90) [14,15]. Hsp90 is a molecular chaperone that assists in stability and function of various mutated or over-expressed proteins responsible for tumor survival and growth. Hsp90 inhibition has shown promise in preclinical cancer studies [14]. Moreover, 17AAG has been an active subject of clinical trials in recent years [16-20]. These findings support our results, which indicate that higher drug activity levels for all of these compounds correspond to better prognosis.

While there are multiple citations supporting our findings for monorden and 17AAG [14-16,18,19,21], far fewer studies promote ikarugamycin in the treatment of cancer. As mentioned, the macrolide class of antibiotics has shown promising effects in reversing drug resistance [13]. Based on these results, ikarugamycin and related compounds may be promising leads as cancer therapeutics. Further biological investigation is warranted.

### Cancer and Toll-like receptors

Bacteria and inflammation due to microbial stimulation of Toll-like receptors have been associated with carcinogenesis and cancer progression [22-26]. Moreover, various studies have shown a relationship between TLR4 activation (the receptor for LPS) and cancer progression [27,28]. The fact that this relationship is not present in some cancers, such as adenocarcinoma (EPSA data not shown) indicates that it is not simply the case that inflammation is associated with cancer progression. Rather, TLR pathway activation appears to have a selective effect in specific cancers. Future bench work will be to probe this hypothesis in a biological system. As an immediate next step, we aim to determine the activity of Toll-like receptors in human diffuse large B cell lymphoma.

Beyond differential prognosis, our approach can provide a starting point for analyzing functionally relevant genes. A number of studies have attempted to define the smallest number of genes that can be used to accurately predict class membership or prognosis in cancer [29,30]. However, these gene sets were selected based on an ability to discriminate between phenotypes, without prior knowledge of gene function or pathway involvement. In contrast, genes in a pathway signature offer additional insight regarding mechanism, since a possible cause of differential expression of those genes is known.

We evaluated a range of uniform *q*-value thresholds to use in determining a pathway signature. A next step will be to use varying thresholds, dependent on the relative degree of perturbation promiscuity. In addition, our current approach to false discovery rate is to compare random correlations to the mean true correlation value. This method tends to miss cases where different subpopulations fall at different extreme ends of the spectrum, since correlation values would be likely to regress to the mean neutral value, easily observed by random chance. A more elegant method with greater sensitivity would be preferable. Finally, we plan to enhance and expand our methods for determining pathway signatures by expanding the scope of arrays on which we base a given profile, looking across all of GEO for relevant studies.

## Conclusion

In conclusion, we have demonstrated the ability to integrate human disease data with publicly available genomic data from experiments performed in humans and in model organisms, even though the data had been generated to answer separate questions. This has enabled us to generate testable hypotheses regarding pathway dysregulation in human disease as well as its effects on patient prognosis. This work demonstrates that we can use publicly available data not only for asking questions about pathways and interactions, but for immediate, clinically relevant hypothesis generation.

## Methods

### Expression-based Pathway Signature Analysis (EPSA) implementation

The EPSA approach involves three main steps. They are illustrated in Figure 1 and described in detail below: 1) definition of pathway signatures from compendium data (A-B); 2) calculation of mean correlations, *r*_1 ... *n*_, between disease profiles and *n *pathway signatures, and the statistical significance thereof (B-C); and 3) survival analysis on a patient dataset using each statistically significant *r *as a risk factor (C-D). Unless otherwise specified, these steps were implemented as scripts in R and microarray data was processed as described by the original authors for each respective dataset.

Pathway signatures were determined by applying the SAM software package (samr package in R, version 1.20) to publicly available microarray datasets [4]. Two-class unpaired comparison was used for comparing activated or treated samples with control samples. The designated groups consisted of three or more replicates in all cases. Sensitivity analysis using the Rosenwald DLBCL dataset [8] showed that choice of *q*-value within a range of 0.01 to 0.1 did not significantly affect results. That is, varying the threshold for false discovery rate and thus the number of genes included in the signature did not alter downstream observations regarding which pathway signatures demonstrated significant correlation, nor which signatures were able to stratify prognosis (not shown). Therefore, a *q*-value threshold for false discovery rate was chosen empirically to include enough genes for statistically significant results.

For the Bild dataset, replicate arrays from each of the five mutation pathways were compared to control GFP-treated tissue profiles. For the Connectivity Map profiles, treated MCF7 cells were compared to untreated MCF7 cells. MCF7 is a human breast adenocarcinoma cell line. We generated pathway signatures using a subset of the 564 total profiles that met the following criteria: (i) Data were generated in MCF7 cells. Although using additional cell types would likely have resulted in a more generic, cell-type-agnostic signature, the need for comparison to different control cell types would have added complexity to the analysis. (ii) At least three replicates were run at a single concentration. Fewer replicates would be less likely to yield reliable results. Finally, for the AfCS mouse B cell dataset, treated versus untreated samples were compared in triplicate and analyzed at the last time point (4 hours). In all cases, for the genes determined to be statistically significant by SAM, median values of log_2_(treated/untreated) were used to represent the ligand signature.

The EPSA score for a ligand was the average correlation between the ligand signature and the corresponding gene expression values across the test profiles. This value was determined using the Spearman rank order correlation coefficient. In order to determine the statistical significance of the resulting correlations, we calculated a *q*-value, or false discovery rate, for the average level of correlation between each ligand and the set of patients [31]. To do this, a selection of *n *genes was chosen randomly 1000 times to represent the signature of the j^th ^ligand, where *n*_*j *_was the number of genes comprising the signature for ligand *j *from the compendium of ligand profiles. The motivation for permutation analysis is to determine the likelihood of obtaining the observed results by random chance. Between two biologically functioning cells, we expect some positive correlation due to the activation of common pathways required for continued cell function. Therefore, we did not randomize by permuting gene labels in the patient samples, as doing so would have resulted in a loss of biologically realistic relationships and an average correlation of close to zero every time. This approach results in increased stringency, and thus increased specificity, when trying to detect statistically high correlations in that the bar for comparison is set higher. Conversely, it increases sensitivity in detection of statistically lower correlations, which facilitates the detection of dysregulated pathways.

For each ligand that had shown a statistically significant level of correlation with a disease dataset, we analyzed degree of correlation as a factor influencing survival using censored survival data. Cox proportional-hazards analysis was performed on the full set of patients, using the *survival *package [32] in R, version 2.3.1 [33]. In addition, patient groups were divided into highest, lowest, and medium level of correlation. Kaplan-Meier survival analysis was performed on the two extreme tertiles, again using R's *survival *package.

### Compendia

The Bild et al. dataset was generated from nine replicate arrays for each of five pathway mutations induced by recombinant adenoviruses in human primary mammary epithelial cell cultures (HMECs), as well as GFP treated HMEC controls [1]. The authors used Affymetrix Human Genome U133 Plus 2.0 Arrays. Pathway signatures for positive controls in mouse cancer models were "regenerated" from genes with human orthologs.

The Connectivity Map dataset consisted of 564 profiles, representing 164 small molecule perturbagens applied in varying concentrations to one or more cell types. Microarray analysis was performed on Affymetrix GeneChip arrays.

The mouse B cell compendium of gene expression data from the Alliance for Cell Signaling has been described [7]. In brief, splenic B cells were purified from mice and cultured with either a ligand or medium alone for 0.5, 1, 2, or 4 hours. With some exceptions, each ligand/time point combination was performed in triplicate. RBC-depleted splenocytes were used as a universal reference for cDNA hybridization. We used the 4 hour time point for EPSA analysis, as it best approximated chronic exposure to a ligand.

### Positive controls

As positive controls, we performed correlation analysis on two datasets using known perturbations of B cells: one in murine cells, one in human. We first evaluated an independent dataset from GEO, GSE1014, with the full set of genes and with varying portions of the genes removed, to simulate loss of signal in translation between species and platform. Dataset GSE1014 consisted of microarray data from mouse B cells stimulated with 6 of the 33 ligands from the original training set. RNA derived from treated cells at the 6 hour time point was hybridized directly with RNA from untreated cells, without a universal reference.

As a human positive control, we used 4 cases from a study in which human B cells were stimulated with a combination of ligands used in the training set [34]. Four separate experiments involved stimulation with AIG and CD40 for 6 hours, AIG and CD40 for 24 hours, AIG alone for 24 hours, and AIG and IL4 for 24 hours. An additional array from this dataset provided information for unstimulated B cells, which we used to calculate the ratio of treated vs. untreated B cells.

## Abbreviations

AB: Activated B cell-like; AfCS: Alliance for Cellular Signaling; AIG: Anti-Ig; DLBCL: Diffuse large B cell lymphoma; CD40: CD40 ligand; CPG: Unmethylated CpG-Containing Oligonucleotide; EPSA: Expression-based Pathway Signature Analysis; GC: Germinal center-like; GEO: Gene Expression Omnibus; GO: Gene Ontology; HMEC: Human mammary epithelial cell; IL4: Interleukin 4; LPS: Lipopolysaccharide; RBC: Red blood cell; SAM: Significance Analysis of Microarrays; TLR4: Toll-like receptor 4; TLR9-Toll-like receptor 9.

## Authors' contributions

JDT performed the research and wrote the manuscript. MGW supplied statistical expertise in methodological development. PJU provided biological and clinical expertise. AJB first proposed the problem to be addressed, and provided input on methodology throughout.

## Pre-publication history

The pre-publication history for this paper can be accessed here:



## Supplementary Material

Additional file 1 **Actual versus randomly permuted correlations using Connectivity Map perturbagen profiles and an ovarian cancer cohort.** This figure shows statistically significant correlations between perturbagen signature profiles and ovarian cancer tumor profiles, compared to randomly generated correlations. Click here for file

Additional file 2 **Correlation values for murine and human positive controls with AfCS compendium ligands.** These panels show the relative correlations between known perturbations and the profiles observed in the AfCS murine dataset. Click here for file

Additional file 3 **Actual versus randomly permuted correlations using AfCS ligand profiles and a DLCBL patient cohort.** Comparison of average correlation of DLBCL patient profiles and murine compendium pathway signatures, versus compendium ligand signatures and randomly permuted signatures. Click here for file

Additional file 4 **Q-values for correlation of Rosenwald data with AfCS compendium ligands.** This graphs illustrates the false discovery rates of the observed correlations between DLBCL profiles and ligands signatures from the AfCS murine dataset. Click here for file
