# Supplementary figures and images for: Expression-based Pathway Signature Analysis (EPSA): Mining publicly available microarray data for insight into human disease
Source: BMC Med Genomics. 2008 Oct 20;1:51. doi: 10.1186/1755-8794-1-51 (PMC2588448; doi:10.1186/1755-8794-1-51)

## Slide 1
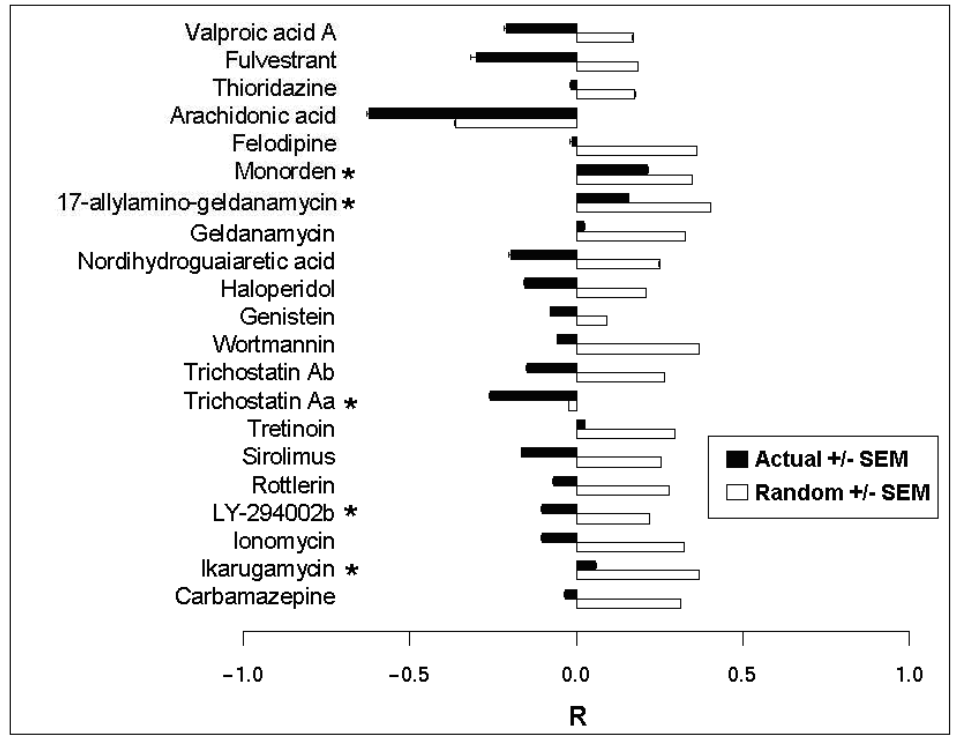

Supplement: Additional file 1 — Actual versus randomly permuted correlations using Connectivity Map perturbagen profiles and an ovarian cancer cohort. This figure shows statistically significant correlations between perturbagen signature profiles and ovarian cancer tumor profiles, compared to randomly generated correlations. [file 1755-8794-1-51-S1.ppt]

## Slide 1
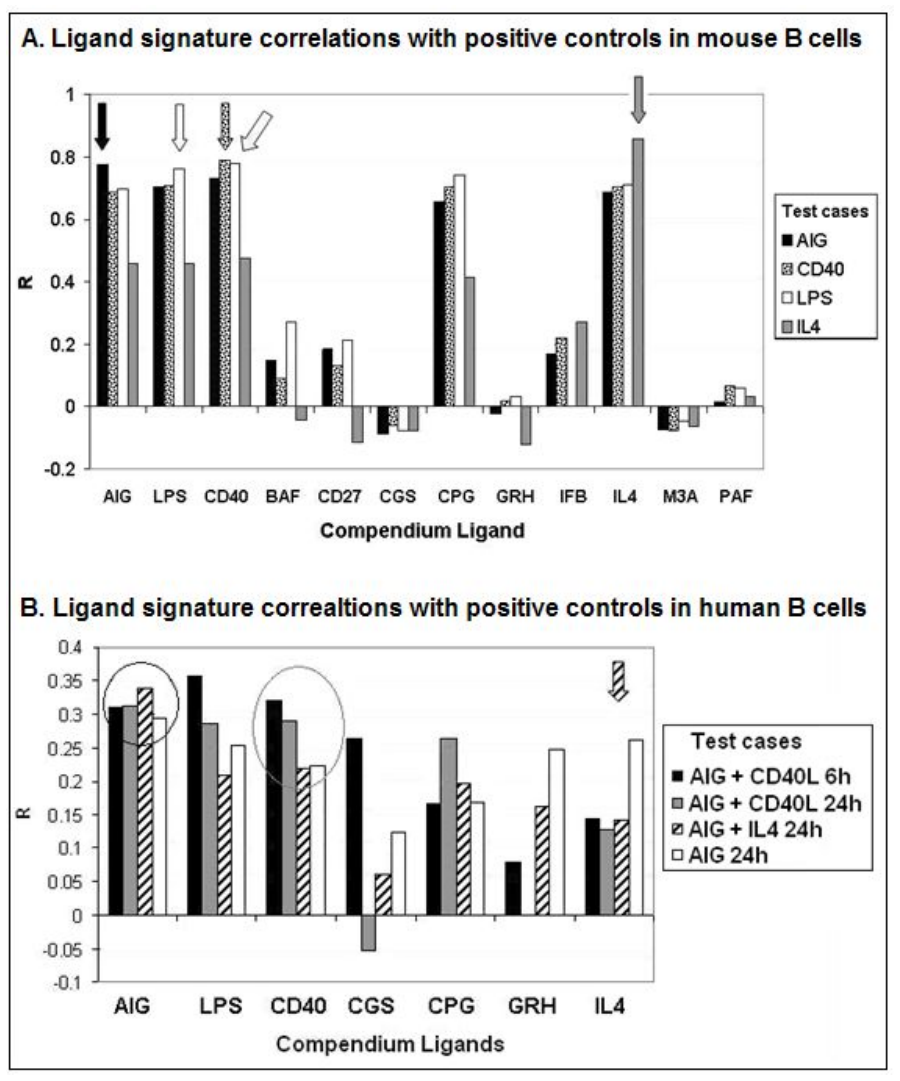

Supplement: Additional file 2 — Correlation values for murine and human positive controls with AfCS compendium ligands. These panels show the relative correlations between known perturbations and the profiles observed in the AfCS murine dataset. [file 1755-8794-1-51-S2.ppt]

## Slide 1
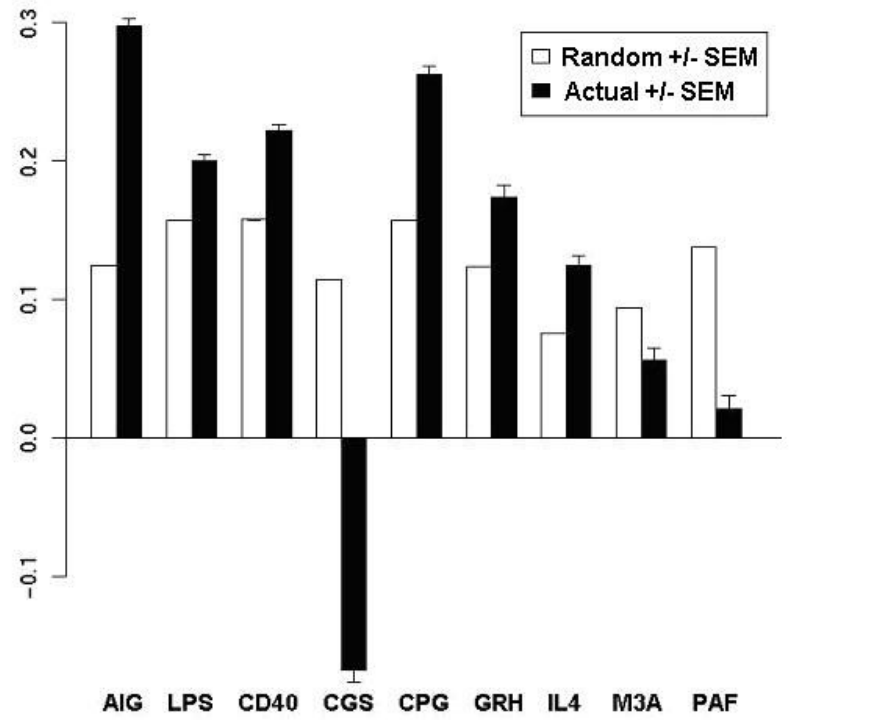

Supplement: Additional file 3 — Actual versus randomly permuted correlations using AfCS ligand profiles and a DLCBL patient cohort. Comparison of average correlation of DLBCL patient profiles and murine compendium pathway signatures, versus compendium ligand signatures and randomly permuted signatures. [file 1755-8794-1-51-S3.ppt]

## Slide 1
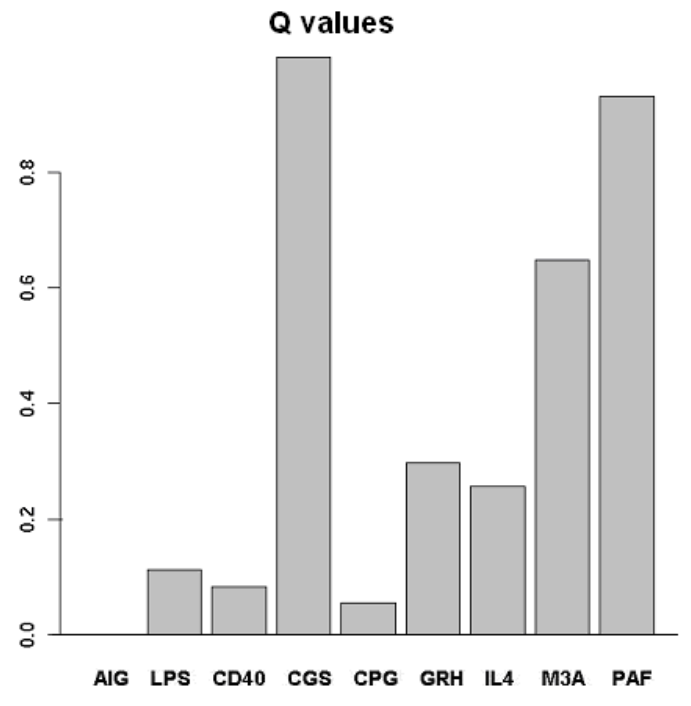

Supplement: Additional file 4 — Q-values for correlation of Rosenwald data with AfCS compendium ligands. This graphs illustrates the false discovery rates of the observed correlations between DLBCL profiles and ligands signatures from the AfCS murine dataset. [file 1755-8794-1-51-S4.ppt]
